# The intrinsic dimension of protein sequence evolution

**DOI:** 10.1371/journal.pcbi.1006767

**Published:** 2019-04-08

**Authors:** Elena Facco, Andrea Pagnani, Elena Tea Russo, Alessandro Laio

**Affiliations:** 1 SISSA, Trieste, Italy; 2 DISAT, Politecnico di Torino, Torino, Italy; 3 IIGM, Italian Institute for Genomic Medicine, Torino, Italy; 4 INFN, Sezione di Torino, Torino, Italy; 5 ICTP, International Centre for Theoretical Physics, Trieste, Italy; Temple University, UNITED STATES

## Abstract

It is well known that, in order to preserve its structure and function, a protein cannot change its sequence at random, but only by mutations occurring preferentially at specific locations. We here investigate quantitatively the amount of variability that is allowed in protein sequence evolution, by computing the intrinsic dimension (ID) of the sequences belonging to a selection of protein families. The ID is a measure of the number of independent directions that evolution can take starting from a given sequence. We find that the ID is practically constant for sequences belonging to the same family, and moreover it is very similar in different families, with values ranging between 6 and 12. These values are significantly smaller than the raw number of amino acids, confirming the importance of correlations between mutations in different sites. However, we demonstrate that correlations are not sufficient to explain the small value of the ID we observe in protein families. Indeed, we show that the ID of a set of protein sequences generated by maximum entropy models, an approach in which correlations are accounted for, is typically significantly larger than the value observed in natural protein families. We further prove that a critical factor to reproduce the natural ID is to take into consideration the phylogeny of sequences.

## Author summary

Protein sequence evolution is an extremely complex process, whose roles are ultimately determined by the necessity of living organisms to adapt to changes in the environment. We here address a fundamental question related with this process: in how many independent directions can a sequence evolve, without compromising the protein capability of folding and of performing its function? We find that the number of these directions is surprisingly small, of 10 or less in most of the families we considered. This property is not correctly accounted for by most of the theoretical model we considered, which predict that sequence evolution can take place in 30-40 independent directions. The only way to accomplish the task of generating low-dimensional sequences is to take into consideration sequence phylogeny.

## Introduction

Protein sequence evolution is an extremely important process in living organisms. During evolution, due to insertions, deletions, substitutions, a sequence can significantly change. Still, and most importantly, three-dimensional structures and functions are conserved, so that protein domains descending from a common ancestor share fundamental common traits; such protein domains form the so-called families. The birth of a new family is a rare event, while existing families are conserved by evolution. Despite the fact that the sequence similarity between members of the same family can be extremely low, by looking at the multiple sequence alignment (MSA) of a protein family one immediately notices patterns. Amino acids in specific columns of the MSA are often conserved, and mutations in different columns are in many cases correlated. This observation is at the very basis of statistical models for assessing the probability that a protein sequence belongs to a family [1] or for predicting the three-dimensional structure of the protein from the MSA [2, 3]. Frequent occurrences of the same amino acid in a column of the MSA together with covariation between different columns suggest that evolution modifies the sequences along a number of directions that is much lower than the bare dimension of the space sampled by randomly substituting amino acids.

We here address a specific question: how many independent directions are explored during evolution in a protein family? This issue can be rephrased in the conceptual framework of Intrinsic Dimension [4]. The Intrinsic dimension (ID) of a data set is defined as the minimum number of parameters needed to describe the data without information loss. Several methods are available to estimate the ID [4]; projective methods aim at representing points onto a lower dimensional space by minimizing an error function [5–7], while fractal methods measure the scaling of the number of points within a certain radius as such radius grows larger [8]. Nearest neighbors-based methods assume that close points are drawn from a uniform distribution and extract models for the statistical distribution of the first distances [4, 9, 10]. We here estimate the ID by TWO-NN [11], since the method is able to deal with density variations in the dataset. This is a fundamental requirement, as the protein sequences belonging to a family are not sampled uniformly: the sequence identity between a sequence and its closest neighbor can vary significantly, even within the same family. Moreover, and possibly more importantly, the TWO-NN estimator provides a criterion of reliability on the ID measure based on the quality of a linear regression.

Key to all the statistical properties of distances, is a notion of metrics on the set of protein sequences. This is a delicate point, since the metric is entangled with the ID estimate. The features that such distance should possess to be suitable for our purposes are essentially two: (i) being a metric (many dissimilarity measures used in bioinformatics do not satisfy the triangular inequality), (ii) to depend only on the pair of sequences it is computed on (thus excluding distances deriving from multiple sequence alignments). Taking into consideration these issues, we develop two notions of distances that we call Modified Hamming Distance, and BLOSUM distance and relate them to the well established Edit distance [12]. By using such distances we are able to compute the dimensions of several Pfam protein families [13], discovering that their IDs range approximately from 6 to 12. These values are robust to the choice of the metric and are correlated, to a certain extent, with the average length of the sequences as well as with the number of different architectures present in the family.

Finally, we study the reliability of artificial generative models for protein sequences from the point of view of the intrinsic dimension. The ID is a complex function of the data, but a meaningful one, and we suggest that it can be employed to assess the goodness of artificial models. We benchmark the capability of reproducing the correct ID of sets of protein sequences generated by HMMER [1], Direct Coupling Analysis [14, 15] and ProteinEvolver [16], an approach which allows simulating protein sequence evolution taking into account phylogenetic history.

## Methods

In this section we describe in detail the procedure to compute the intrinsic dimension of protein families. In particular, we address the following issues:

- Definition of a distance between sequences
- ID computation

The datasets we analyze are obtained by downloading the FASTA sequences of full families from the Pfam website [17]. Since we are interested in estimating the intrinsic dimension of protein sequence evolution at intermediate evolutionary distances, as a preliminary step we filter out correlated entries by means of CD-HIT [18], with a threshold of 80% of sequence similarity.

### Definition of a distance between sequences

Defining a “good” distance between points is a crucial step to compute the intrinsic dimension of a dataset. In general under different metrics the ID can change. If the dataset is not representable in terms of coordinates (as in the case of protein sequences) the space itself is fully described by a set of pairwise distances. It is therefore of fundamental importance to analyze the relationship between the notion of distance employed and the resulting intrinsic dimension. In a formal context two metrics *d* and $\tilde{d}$ are said to be *equivalent* [19] if and only if there exists a finite positive constant *C* such that $\frac{1}{C}d\left(x,y\right)\leq \tilde{d}\left(x,y\right)\leq Cd\left(x,y\right)$. In practice, when comparing two notions of metrics it is possible to look at their correlation to infer their equivalence. Indeed, the intrinsic dimension is unchanged when the metric is altered to an equivalent metric [19]. Thus, even if we have at our disposal only the finite set of pair distances defined on a set of sequences, we expect that in the case of a good correlation between the distances obtained with different metrics the ID will be unchanged; this means that the intrinsic dimension is not only an attribute of a notion of distance, but rather of a class of distances associated to each other in terms of correlation.

Several methods are available in the literature to estimate pairwise sequence distances and similarities [20]. A definition of distance has to fulfill some fundamental requirements in order to describe a set of relationships between sequences where the ID estimation is well-posed. First of all we want our definition of dissimilarity *D* to resemble as much as possible a metric, meaning it should be non-negative, equal to zero only for identical sequences, symmetric, and it should satisfy the Triangular Inequality (TI). Another requisite we prescribe is that the notion of dissimilarity between two sequences depends only on the sequences themselves. Some of the well-known notions of distances, for example the Hamming distance [21], are based on a Multiple Sequence Alignment (MSA); in a MSA the match between two residues in two different sequences does not depend only on the two sequences, but on all the sequences used to derive the MSA: in this way the distance between two entries builds upon all the other sequences in the set, and a simple operation as adding new entries to the dataset could change the overall distribution of distances. For this reason our definitions of dissimilarity will rely only on pairwise sequence alignments. In the following we describe three notions of distances that fulfill the requirements enumerated above.

#### Modified Hamming distance

This measure of distance is based on pairwise alignment between sequences by means of BLAST [22]. Since the TWO-NN intrinsic dimension estimator requires finding only the closest and the second closest nearest neighbors, only entries with an E-value lower than 10 are retained. If for sequences *s*_1_ and *s*_2_ two (or more) relevant MSPs, or alignments, are found, the one with lowest E-value is retained. Note that, due to its heuristic nature, BLAST is not symmetric, meaning that in principle, it could align differently an ordered pair of sequences (*A*, *B*) from the reversed (*B*, *A*). In this case the best (in terms of E-value) of the two alignments is retained. We define the Modified Hamming distance as:

$$d_{MH}\left(s_{1},s_{2}\right)=\{\begin{array}{cc} \frac{m-L\times \frac{P}{100}}{m} & \text{if}\;\text{E-value}(s_{1},s_{2})<10.\\ 10 & \text{otherwise} \end{array}$$ (1)

Here by *P* we denote the percent identity of the best alignment between *s*_1_ and *s*_2_, by E-value(*s*_1_, *s*_2_) we indicate its E-value, while *m* = max{*m*_1_, *m*_2_} is the maximum between the two sequences lengths and *L* is the alignment length. In words, this corresponds to scoring matches as 0, mismatches as 1, counting the not aligned amino acids as mismatches and dividing by the maximum length of the two sequences. Note that by definition the distances lower than 10, that is to say those actually deriving from an alignment, are bounded above by 1.

We analyzed all significant triplets of sequences in a number of different PfamA families to count the percent of violations of the Triangular Inequality. The results obtained confirm that Modified Hamming is susceptible to TI violations but in such a small amount that it can be considered a metric. For instance in the case of PfamA family DnaJ the number of violations is 645 over a number of proper triplets of 69 × 10^7^, thus only ∼ 9 × 10^−5^% of the entries are involved.

#### BLOSUM distance

We also defined a variation of the Modified Hamming distance that instead of scoring the mismatches as 1 assigns them a score according to a BLOSUM matrix. The computation employs the bitscore of pairwise alignments (again part of the BLAST output), that is based on the score matrix BLOSUM62. Given two sequences *s*_1_ and *s*_2_ we define the BLOSUM distance *d*_*BL*_ as:

$$d_{BL}\left(s_{1},s_{2}\right)=\{\begin{array}{cc} \frac{M-S}{M} & \text{if}\;\text{E-value}(s_{1},s_{2})<10.\\ 10 & \text{otherwise} \end{array}$$ (2)

Here *M* is the maximum bit score between *s*_1_ against itself and *s*_2_ against itself, and *S* is the bitscore of the best alignment. We empirically verified that *d*_*BL*_ is, to a good approximation, a metric.

In the same framework, we also considered a distance *d*_*SD*_ capable of capturing sequence divergence for short evolutionary times. At this scope, we performed sequence alignments using the substitution matrix from [23], which takes into account the evolutionary likelihood of a substitution from a codon model. We then followed the procedure in [24] to obtain an amino acid substitution matrix, at a reference sequence identity of 96%. We estimated the distance by Eq 2, aligning the sequences and computing the the scores M and S with this matrix. As shown in S1 Fig, the modified Hamming distance *d*_*MH*_, the BLOSUM distance *d*_*BL*_ and the distance *d*_*SD*_ are well correlated at short distances.

#### Normalized edit/Levinstein distance

In information theory the Levenshtein (or Edit) distance is a string metric for measuring the difference between two sequences (see [25]). Informally, the Levenshtein distance between two words is the minimum number of single character edits (insertions, deletions or substitutions) required to change one word into the other. The Edit distance in its basic formulation is a true metric, and the TI can be formally proved. Instead of using plain Edit we normalize it by the average length of the sequences, to apply a correction to the fact that it is easier for short sequences to present fewer mismatches. The normalized Edit can be also considered a metric, and is well correlated with *d*_*MH*_ at short distances, as displayed in S1 Fig). In the following we show that, as expected, these three notions of distance lead to consistent ID measures.

Finally we considered other definitions of dissimilarity between sequences, namely the p distance, the Kimura distance and the Jukes Cantor distance [20, 26]. We verified that none of these distances is well correlated with *d*_*MH*_, mainly for the reason that they are computed on sequences aligned in an MSA. These dissimilarity measures do not depend only on pairs of sequences, and therefore, based on the considerations above, are not considered in our analysis.

### ID computation

In this section we describe a procedure to estimate the intrinsic dimension of protein families where distances between sequences are defined according to *d*_*MH*_. Even if *d*_*MH*_ is to a good approximation a metric, and thus a basic requirement of TWO-NN is fulfilled, it has an upper bound *u* = 1 that may induce artificial inhomogeneities in the space. TWO-NN method [11] is rooted on the computation, for each point **x** in the dataset, of its first and second nearest neighbors distances *r*_1_ and *r*_2_; the ratios $\mu \left(x\right)≐\frac{r_{2}\left(x\right)}{r_{1}\left(x\right)}$ are collected to provide a measure of ID by a fitting procedure; if the hypothesis of the method are fulfilled, the set *S* given by *S* = {(log(*μ*_*i*_), − log(1 − *F*^*emp*^(*μ*_*i*_))) | *i* = 1, …, *N*}, where *F*^*emp*^ defines the empirical cumulate, is well fitted by a straight line whose slope corresponds to the intrinsic dimension of the dataset.

If we blindly apply TWO-NN to a dataset of *d*_*MH*_ distances obtained on Pfam family DnaJ we obtain a curved *S* set, that cannot be fitted by a straight line, indicating that TWO-NN cannot be applied in a straightforward fashion. We verified that this effect is a consequence of the upper bound interference. So if we could restrict our measure to neighborhoods whose radius *r* is relatively smaller than 1., we should be able to wash away the effects of the upper bound.

To implement this idea, we proceed as follows: let *A* be the set of *μ* values obtained on the whole dataset of *d*_*MH*_ distances. For different values of $\bar{r}$ we retain sets of *μ* values $A_{\bar{r}}\subset A$ such that a value $\mu =\frac{r_{2}}{r_{1}}$ belongs to $A_{\bar{r}}$ if and only if $r_{2}\leq \bar{r}$. In symbols:

$$A_{\bar{r}}≐\left\{\;\mu \in A\;∥\;r_{2}<\bar{r}\;\right\}.$$ (3)

**Fig 1:**
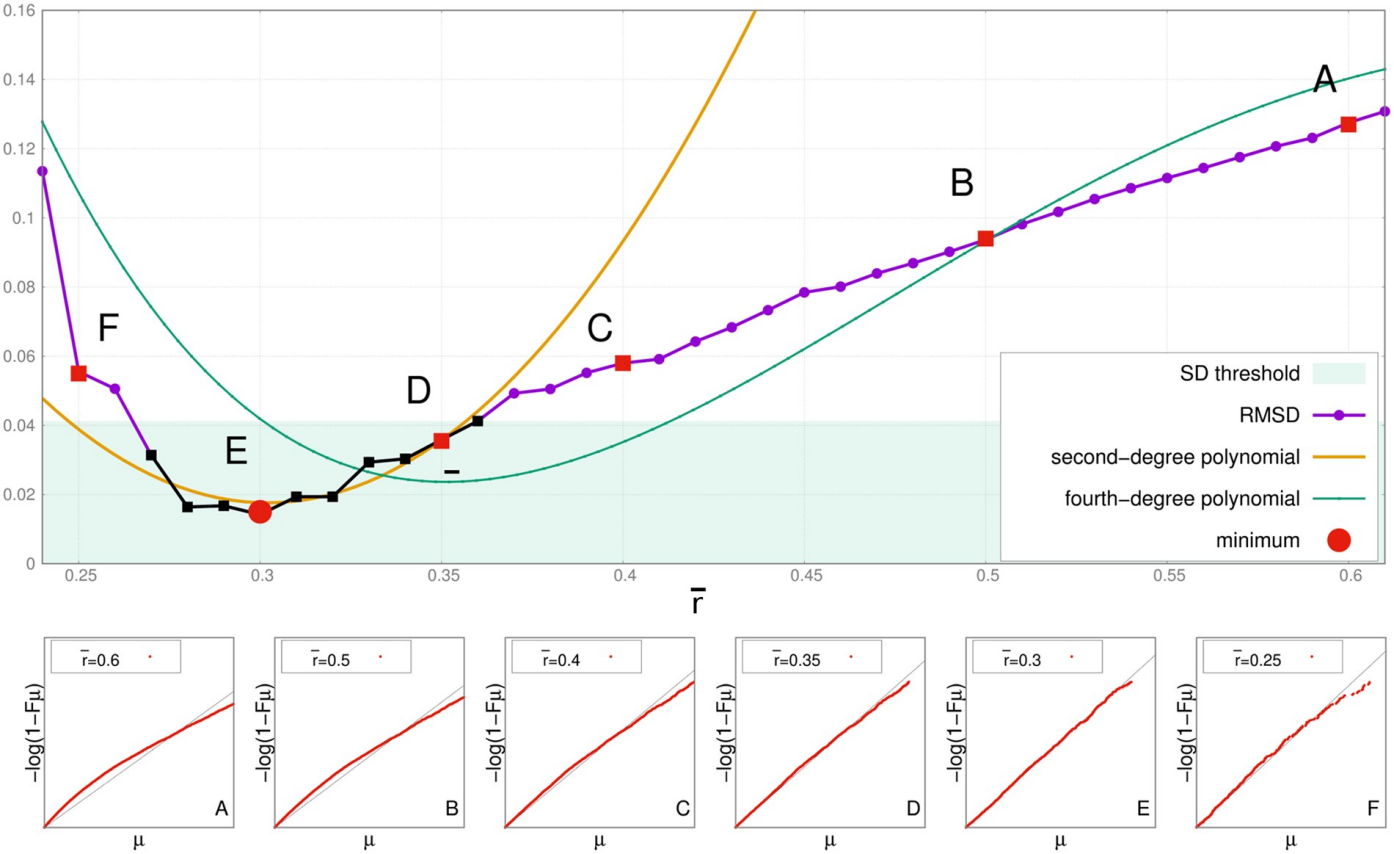
Technical procedure to locate the threshold value minimizing the RMSD. Upper Panel: RMSD of the sets $S_{\bar{r}}$ for different values of $\bar{r}$. The minimum of the RMSD is obtained for $\bar{r}=0.3$ (point E). In green we show the four-degree polynomial fitting the RMSD, with standard deviation SD. The RMSD values falling below the minimum of such polynomial plus SD (green area) are plausible RMSD minima (highlighted in black). We fit these plausible RMSD minima by a second-degree polynomial (yellow curve). The argument of the minimum of such polynomial is considered as the putative value minimizing the RMSD. Panel A: sets $S_{\bar{r}}$ for $\bar{r}=0.6$. Panel B: same as A for $\bar{r}=0.5$. Panel C: same as A for $\bar{r}=0.4$. Panel D: same as A for $\bar{r}=0.35$. Panel E: same as A for $\bar{r}=0.3$. Panel F: same as A for $\bar{r}=0.25$. The *S*_*set*_ here is well fitted by a straight line, while the others show a curvature.

We then estimate the ID using TWO-NN only for the *μ* belonging to $A_{\bar{r}}$. To find out the optimal value of $\bar{r}$, for each value of $\bar{r}=0.25,0.26,\ldots ,0.6$ we compute the Root-Mean-Square Deviation (RMSD) of the fit of $S_{\bar{r}}$ to a straight line. The best choice for $\bar{r}$ is ideally the one minimizing the RMSD. The problem is that the RMSD around the minimum value fluctuates, therefore we set up a procedure to find such minimum together with an estimate of the error on the ID measure. First, we fit the points of the RMSD by a fourth-degree polynomial (cfr. Fig 1) in order to roughly locate the minimum. By performing the fit, we also compute the standard deviation (SD) of the data from the fitted curve. We then consider as putative minima all the data points falling within SD from the minimum of the polynomial. The optimal $\bar{r}$ is obtained by fitting a quadratic curve to this points, and finding its minimum argument, thus refining the previous computation. The error *ϵ*_*d*_ on the ID measure is estimated by the difference between the highest and the lowest value of the ID among the ones obtained for all the putative minima. In Fig 1, upper Panel, we plot the RMSD as a function of $\bar{r}$ for the PfamA family PF00226. In the bottom panels we plot the corresponding sets $S_{\bar{r}}$. It is clear that as $\bar{r}$ decreases towards the value $\bar{r}=0.3$ the pronounced curvature that is visible in the set *S*_0.6_ starts to diminish, and *S*_0.3_ can be well fitted by a straight line. For lower values of $\bar{r}$ the curvature becomes visible again as we begin to see the effects of a new boundary, this time represented by $\bar{r}$ itself. We see that the hypotheses underlying TWO-NN are fulfilled within a range of $\bar{r}$ in which *r*_2_ is far enough from the boundary, yet $\bar{r}$ is large enough not to influence the *μ* distribution. In the case of the example, the minimum of the RMSD is well defined and located at a value of 0.3. the ID corresponding to $\bar{r}=0.3$ is ∼ 9.

Wrapping up, the procedure to compute the intrinsic dimension of protein families is the following:

- Cluster sequences by sequence similarity through CD-HIT in order to obtain a reduced dataset where sequences that are too similar are excluded. This allows estimating the ID on an intermediate scale of sequence identity.
- Use BLAST to perform pairwise alignments and compute the Modified Hamming distance between sequences. The result is a sparse matrix where very high distances are not measured, but set to a default value. This choice speeds up the computation of distances, but introduces a boundary effect.
- Cure the boundary effects by computing the ID on a reduced dataset whose second nearest neighbors are “far enough” from the boundary, that is to say they are below a threshold $\bar{r}$. To find the optimal upper bound $\bar{r}$ try different values and choose the value corresponding to a set $S_{\bar{r}}$ that minimizes the RMSD of the fit to a line.

We verified that, in accordance with the considerations about correlation and equivalence we made above, the ID obtained by this procedure is consistent in the class of equivalent metrics encompassing the Edit distance, the Modified Hamming distance and the BLOSUM distance. In the case of Pfam family PUA the ID computed with the three different metrics is 11.2 for the Modified Hamming distance, 9.9 for the BLOSUM distance and 8.8 for the EDIT distance. This variation is of the order of magnitude of the estimated error on the ID. We also considered the distance obtained by using the substitution matrix from [23], which takes into account the evolutionary likelihood of a substitution from a codon model (see Methods). The ID estimated with this distance is ∼ 4.5, somehow lower. This discrepancy is relatively small, taking into account that this distance is built on a completely different principle with respect to the other three distances: indeed, it is designed to capture the quantified sequence divergence for short evolutionary times, while the other are more appropriate for describing divergence at intermediate or large times.

In the next section we apply the methods discussed above to the analysis of the ID of a set of Pfam families.

## Results and discussion

### Computing the intrinsic dimension of Pfam protein families

**Fig 2:**
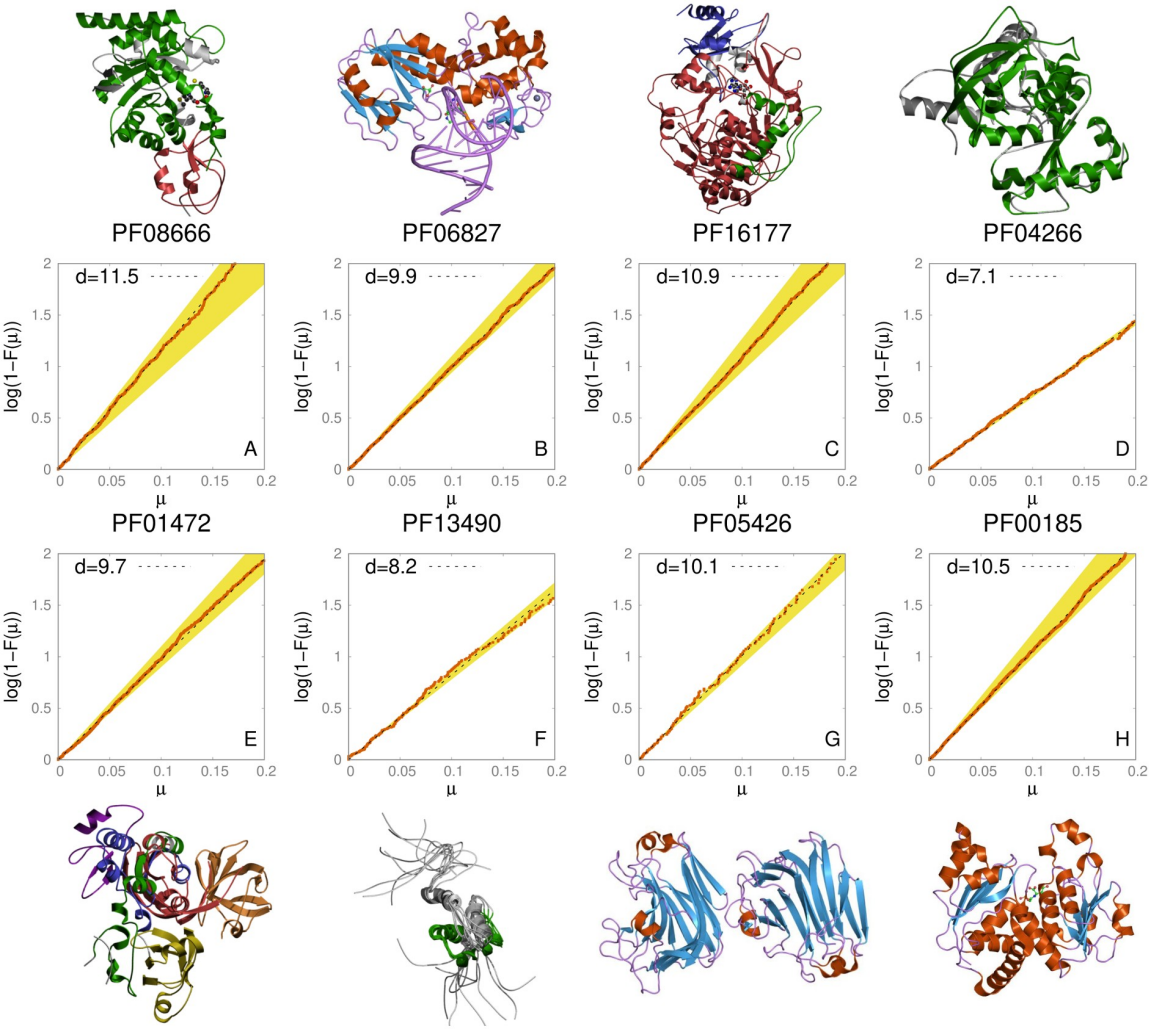
S set and estimated ID for a selection of Pfam A families. Panel A: we display the S set and the intrinsic dimension for PFAM domains PF08666 and the relative pdb structure. Panel B: same as A for PFAM domain PF06827. Panel C: same as A for PFAM domain PF16177. Panel D: same as A for PFAM domain PF04266. Panel E: same as A for PFAM domain PF01472. Panel F: same as A for PFAM domain PF13490. Panel G: same as A for PFAM domain PF05426. Panel H: same as A for PFAM domain PF00185. The pdb structures show that the results do not seem to depend on the structural characteristics that, among the chosen families, turn out to be highly heterogeneous.

We analyzed several Pfam families belonging to different Pfam clans in order to explore a wide range of cases. The procedure explained in the previous section was first applied on a selection of families of Pfam release 31.0 enumerated in Table 1. The families are extracted from clans that are very different from each other: clan CL0489 for instance includes antifreeze proteins, while clan CL0378 consists of enzymes including luciferase. In Fig 2 we summarize the results obtained on some of these families.

**Table 1:** Intrinsic dimension, lenght of the seed alignment, architecture entropy and error on the ID measure *ϵ*_*d*_ for 27 Pfam familes in Pfam notation.

Family	ID	Hmm length	Entropy	*ϵ*_*d*_
PF04266	7.2	107	0.17	0.16
PF01472	9.7	74	1.65	1.93
PF14306	11.	159	1.39	0.87
PF07786	8.3	223	0.38	1.41
PF02009	10.2	321	0.48	1.72
PF16976	8.4	116	0.78	1.67
PF02886	7.4	238	1.11	4.7
PF08666	11.6	63	1.61	3.57
PF13144	7.6	196	0.12	1.72
PF16177	10.9	55	0.61	2.87
PF04326	9.1	122	1.53	4.83
PF01918	8.3	67	0.16	1.12
PF01177	10.3	224	0.10	1.80
PF00471	7.3	47	0.01	0.27
PF01020	5.6	50	0.99	0.98
PF05170	8.	608	1.34	4.71
PF00185	10.5	157	0.43	2.42
PF13502	6.8	229	1.41	3.91
PF06827	9.9	30	0.89	1.59
PF13116	6.6	289	0.78	1.80
PF00226	9.1	63	2.64	0.75
PF08613	8.4	161	0.44	1.48
PF04357	7.1	383	0.44	1.35
PF00382	9.1	71	1.74	2.70
PF04080	8.8	255	0.39	0.67
PF00049	5.7	85	0.66	1.53
PF05426	10.1	272	0.92	0.79

First of all we note that in all the cases the reduced S-sets are very well fitted by a straight line, meaning that the procedure introduced in this work leads to a well defined intrinsic dimension. More precisely, this property implies that the ID is practically constant for sequences belonging to the same family [11]. The slopes of these straight lines, corresponding to the dimensions of the families, span from a value of around 6 to around 12; these values are quite similar and low relatively to the dimension of the embedding space: in fact, representing all the sequences of a family in a vector space would require in principle *L* coordinates, where *L* is the maximum sequence length in the family; *L* is normally of the order of at least 300 in all the cases displayed in Table 1. If we look at the sequence similarity within a family, we find entries sharing only 20% of the amino acids so that the number of mutations observed in a family is enormous. The low ID of the manifold containing the sequences can be interpreted in terms of allowance for mutations: the evolutionary pressure results in a lack of variations at specific positions and in correlated variations across different positions, both restricting the number of degrees of freedom. These results are consistent with the low dimension of 4 found in [27] for 6651 encoding for voltage sensor domains. In this case, though, the ID is computed by means of the Hamming distance on sequences aligned in a MSA. As we discussed above, this measure of similarity is not, strictly speaking, a metric, since its value for a pair of sequences depends on all the other sequences in the MSA. The intrinsic dimension is in principle different in different families, and this is what we observe in our measures. The natural question arising at this point is what determines a specific value of the intrinsic dimension.

In order to address this question, we first tested the dependence of the ID on the length of the Hidden Markov Model (HMM) of the family. A possible guess is that the longer is the length of the HMM, the larger the ID, as a longer HMM could allow for larger variability across the family. We study the correlation between the HMM length and the ID of the Pfam families listed in Table 1. This analysis is to be carried out carefully, since the measure on the ID is affected by an error that could, in principle, hide the correlation signal. To deal with such error, we retain only the 10 families with smaller error on the ID measure. On this subset of families the correlation between the ID and the seed length is rather small, with a Pearson coefficient of ∼ 0.2. This indicates that the ID value is only slightly correlated with the typical length of the sequences belonging to a family.

We also investigated the possibility that the ID is correlated with the number of domain architectures in the family. Some families show a great variability of architectures while others are well represented by a single one. It is plausible that families encompassing a wider number of different architectures have a greater variability and thus a larger ID. To test this hypothesis, we examined the Pearson correlation between the intrinsic dimension and the entropy of the distribution of domain architectures across the family, defined as:

$$S=-\sum_{a}\frac{N_{a}}{N}\text{log}(\frac{N_{a}}{N}).$$

Here *N* is the total number of sequences in the family and *N*_*a*_ is the number of sequences in the family associated with a given architecture *a*. Again, we compute the correlation on the first 10 families in which the ID is computed with smaller error. In this case the computed Pearson coefficient is ∼ 0.3. The analysis suggests that part of the variability of the ID in different families can be ascribed to the differences in the length of their typical sequence and to the number of architectures included in the family.

### Estimating the ID of artificially generated protein sequences

We finally studied the reliability of artificial generative models for protein sequences from the point of view of the intrinsic dimension; we have seen that the dimension of protein families is relatively low; this is likely to be due to the constraints imposed by the three dimensional structure, that narrow to a great extent the allowed mutations. The aim of statistical models of protein sequence evolution is to unveil the statistical constraints underlying the variability of sequences within a family, as well as to link this information to the conservation of biological structures and functions [14]. Once the statistical model has been set up, it could in principle be used in an inverse fashion, to generate sequences. A virtually perfect generative model should be able to reproduce the salient constraints acting on a family, and in particular the low ID. Over the last decade a number of global statistical inference approaches has been developed [1–3, 28–35]. In all the cases, the starting point is a Multiple Sequence Alignment (MSA) on a protein family. MSAs are rectangular matrices $A=\{a_{i}^{\nu }|i=1,\ldots ,L,\nu =1,\ldots ,M\}$, where *M* is the number of sequences, *L* the length of the alignment and $a_{i}^{\nu }$ is either one of the 20 amino acids or a gap “-” standing for insertions or deletions (we stress that in this way gaps are considered on a par with any of the 20 amino acids, in accordance with standard practice). Thus, each line in a MSA corresponds to a protein sequence (*a*_1_, …, *a*_*L*_). A viable assumption for modeling the MSA is that it constitutes a sample of a Boltzmann distribution in the space of sequences:

$$P\left(a_{1},\ldots ,a_{L}\right)=\frac{1}{Z}\text{exp}\left\{-H\left(a_{1},\ldots ,a_{L}\right)\right\}\;\;\;,$$ (4)

where Z is a normalization constant. The key point of model inference is the reconstruction of the form, together with its specific coefficients, of the Hamiltonian H in the exponent of Eq 4. For instance, if the purpose is to reproduce exactly the first empirical moments computed from the MSA, as in the case of [1], H will take the shape:

$$H\left(a_{1},\ldots ,a_{L}\right)=-\sum_{i=1}^{L}h_{i}\left(a_{i}\right),$$ (5)

where *h*_*i*_(*a*_*i*_) = log *f*_*i*_(*a*_*i*_) and *f*_*i*_(*a*_*i*_) is the empirical frequency count computed from the reference MSA for the occurrence of amino-acid *a*_*i*_ at alignment position *i*. Increasing the level of complexity of the generative model, we considered artificial protein families generated by hmmemit (from the HMMER suite [1]), where the emission probabilities are those of the hidden Markov model which produced the MSA. Beyond reproducing the local frequency counts as in (5), this strategy allows taking into account the gap-gap non-local correlation structure generated by the frequent appearance of repeated gap stretches in specific parts of the alignment. Finally, as the most accurate generative model, we use Direct Coupling Analysis (DCA), that aims at reproducing also the empirical distribution of second moments, i.e. the empirical pair frequency counts *f*_*ij*_(*a*_*i*_, *a*_*j*_). These are obtained by counting the co-occurrence of amino acids *a*_*i*_, *a*_*j*_ at position *i*, *j* in the MSA. Maximum entropy modeling dictates for H the following functional form:

$$H\left(a_{1},\ldots ,a_{L}\right)=-\sum_{1\leq i<j\leq L}J_{ij}\left(a_{i},a_{j}\right)-\sum_{i=1}^{L}h_{i}\left(a_{i}\right).$$ (6)

The model parameters encoded in the interaction matrix *J*_*ij*_(*a*_*i*_, *a*_*j*_), and in the biases *h*_*i*_(*a*_*i*_) can be estimated, for instance, by means of Boltzmann learning (BL) [36, 37]. The gaps are taken care of following the standard procedure of DCA, i.e. considering gaps as the 21st amino acid. Their frequencies and length are therefore by construction consistent with the corresponding distributions observed in the MSA.

**Fig 3:**
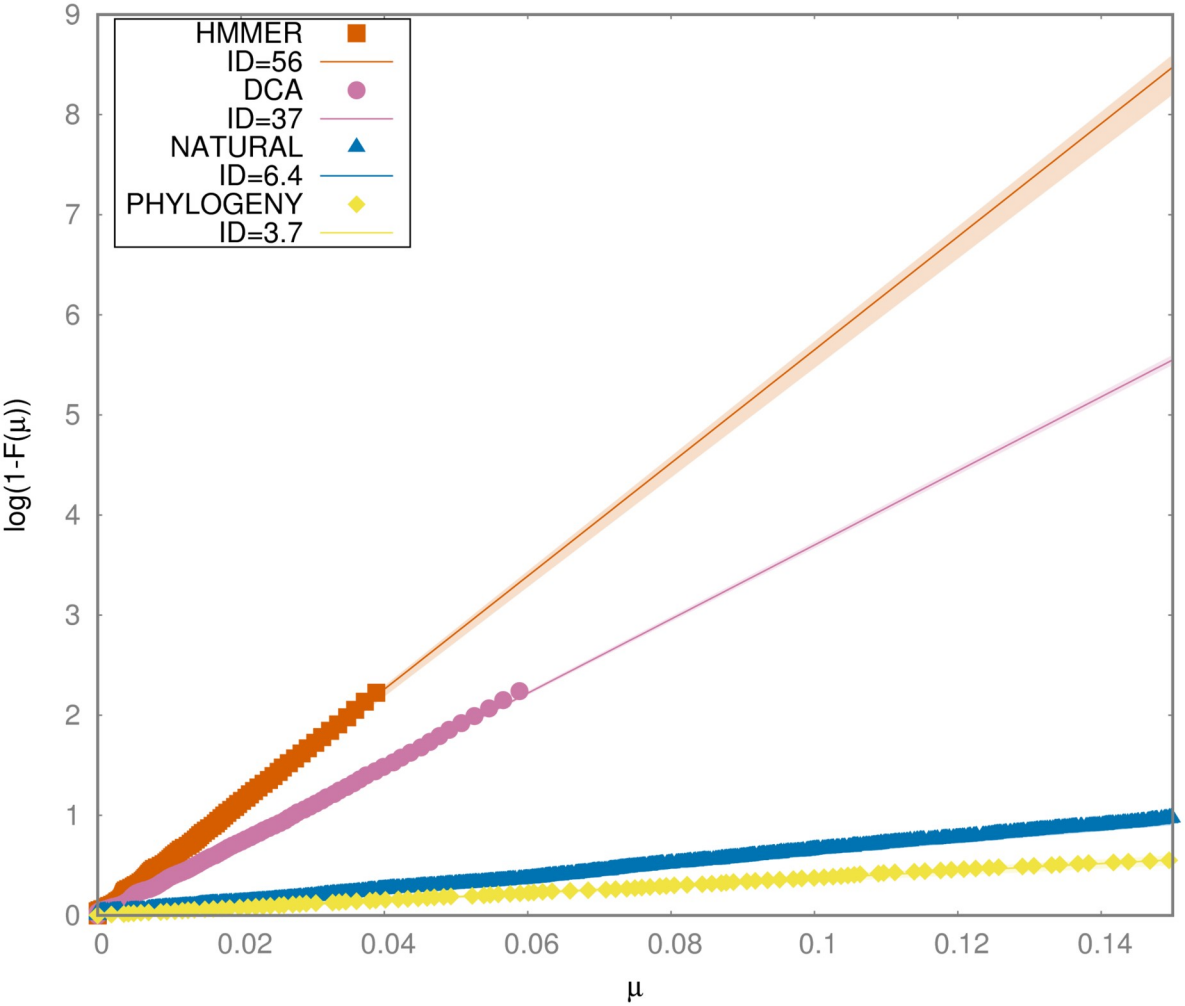
S set and fitting for generated dataset. S set and fitting lines together with the corresponding error on the fit (filled area) for sequences generated with HMMER [1], ACE [37], with the model by Arenas *et al* [16] introducing the phylogeny factor and for natural ones, in the case of Pfam family PF00076.

The interest of the latter model lies in the fact that strongest pairwise couplings turn out to provide accurate prediction of contacts between residues, thus enabling protein-structure prediction. It is then natural to expect that artificial sequences generated from 6 can mirror other characteristics of natural sequences, which are not explicitly fitted by the model. To test the validity of models 5 and 6 with respect to the ID we study the case of Pfam family PF00076. In Fig 3 we analyze the ID of sequences generated with HMMER [1] and DCA, and compare it to the ID of natural ones. The intrinsic dimension of sequences generated with HMMER [1] is the highest, with a value of ∼ 56 (with lowest ID within the error bars equal to 52), since the constraints related to covariation are not taken into account by construction. Sequences generated by means of DCA have a lower ID, as a consequence of the couplings, but the dimension is ∼37 (with lowest ID within the error bars equal to 27), still high with respect to the natural ones; in fact, according to the other Pfam families we analyzed, natural sequences from family PF00076 lie on a manifold with ID ∼ 6.

Taken together, these observations suggest that even if DCA is able to provide accurate predictions of contacts between residues, and thus to give insight into protein structure, yet it is not able to reproduce the ID of natural protein families. To do so, probably, pairwise couplings and local fields are not sufficient, and a more complex model has to be considered. From this point of view, the ID indicator turns out to be a severe and stringent touchstone for artificial sequence generation. To test this hypothesis, we simulated protein sequence evolution by the model introduced Arenas *et al* [16]. This approach allows taking into account the structure of the native state, and phylogenetic history at the same time. This problem was recently raised in an interesting paper by Qin *et. al* [38], where the naive use of of DCA from MSAs was criticized due to the neglected confounding effect induced by phylogenetic branching.

We first performed the dynamics using as a reference the chain A of the structure 1g2e from the Protein Data Bank. This chain is one of the representative structures of Pfam family PF00076. We then selected at random 1000 sequences from the same family, and we built a phylogenetic tree using FastTree v2.1 [39], an approximately-maximum-likelihood phylogenetic trees reconstruction from alignments of nucleotide or protein sequences. We ran ProteinEvolver [16], generating 5 replicates with the default settings provided in the distribution (the input files are produced as shown in S1 File). In this manner, we were able to generate a set of 5000 sequences that we analyzed with the standard procedure.

The result is shown in Fig 3: the ID of the set of sequences is ∼ 4, slightly lower than the true value for this family ∼ 6 but of the same order of magnitude. We verified that this result is robust with respect to changes of the parameters of the model (size of the phylogenetic tree, substitution model, sequence of the seed, etc.). In particular, we repeated the procedure starting from ten different sequences from the same family PF00076: A0A0P7BDN8_9HYPO/316-385, A0YL37_LYNSP/3-73, A0A0K8LDY3_9EURO/366-436, A0A0K3AU17_CAEEL/271-341, A4RRZ6_OSTLU/23-94, Q86BL5_DROME/654-724, A0A0V1HP59_9BILA/14-84, F1QWP0_DANRE/26-96, E2RGA8_CANLF/456-526, A0A0D1X4N1_9EURO/250-320. These sequence differ significantly in their sequence. For each of these initial seeds we repeated the whole procedure. The ID in each case differs by maximum 0.3 from the one generated starting from the sequence of 1g2e. This analysis demonstrates that taking into account phylogeny drastically reduces the dataset ID, thus reproducing the characteristic low intrinsic dimension peculiar to natural sequences.

### Concluding remarks

In this work we study the intrinsic dimension of samples of protein sequences belonging to the same families. This value is a measure of the number of independent directions that evolution can take starting from any given sequence. A key point to properly address the problem is defining an appropriate distance between data points, namely the protein sequences. Many measures of sequence similarity introduced in the literature cannot be used in this context, either because they do not define a metric (not even approximately) or because their value does not depend only on pairs of sequences. Remarkably, the three measures of sequence similarity that can be considered appropriate distance measures, the Modified Hamming distance, the BLOSUM distance, and the Edit distance, are (roughly) equivalent [19], and lead to approximately the same value of intrinsic dimension. While we cannot exclude that other viable distance measures exist, which are not equivalent to the ones we consider here, this result implies that the intrinsic dimension we find is rather robust with respect to the exact choice of the metric, within the same class of equivalence.

By exploiting the properties of the TWO-NN estimator [11], we find that the intrinsic dimension is approximately constant within a family. This claim is non-trivial: in principle, one could expect the ID to vary in different branches of the evolutionary tree, but our results suggest that this is not the case. Empirically, we find that the intrinsic dimension varies within 6 and 12 among the families we considered, and we find hints that this variation can be at least partially ascribed to the average length of the protein sequence, and to the number of architectures that are present in a family. These values are remarkably small, especially because we considered only sequences differing by at least 20% of residues. As a consequence, if one observes ∼ 60 mutations in a sequence of 300 amino acids, typically these mutations are strongly correlated, and can be accurately described by a “basis” of dimension between 6 and 12. The low ID can be at least partially interpreted in terms of allowance for mutations: the evolutionary pressure results in a lack of variations at specific positions and in correlated variations across different positions, both restricting the number of degrees of freedom of the sequences. However, we find that the value of the ID is not reproduced by Direct Coupling Analysis, an approach that models these effects by a pairwise Potts-like Hamiltonian defined on the sequence space. Indeed, the value of the ID measured on sets of artificial sequences generated by DCA is several times larger than the value observed in natural sequences, even though DCA reproduces exactly the pair probability of observing two amino acids in two different sites (see also S2 Fig). Given the recent interest in using maximum entropy models to generate *in silico* functional protein sequences [40–44], we believe that ID analysis provides a very stringent test to assess the accuracy of the generative model to reproduce natural sequences belonging to a given protein family. We finally demonstrate that taking into consideration phylogeny in protein sequence evolution [16] implies a drastic reduction in the ID, to values that are close to those observed in the natural sequences. This indicates that the phylogenetic structure of the mutation history is essential for generating ensembles of structures with an amount of correlation consistent with observations.

## Supporting information

S1 Fig Correlation plots in the case of pfamA family PUA. (A) Correlation plot between *d*_*BL*_ and normalized Edit distances (B) Correlation plot between *d*_*BL*_ and *d*_*MH*_ distances. (C) Correlation plot between *d*_*SD*_ and *d*_*MH*_ distances. The four distances are correlated, especially at low values.
(TIF)

S2 FigHistograms of Hamming distances.Artificial sequences should in principle be indistinguishable from natural ones. One of the characteristics they are supposed to share, tested in [14], is the set of Hamming distances of sequences to the consensus sequence $\left(a_{1}^{*},\ldots ,a_{L}^{*}\right)$, defined by the most frequent amino acids $a_{i}^{*}={\text{argmax}}_{a}f_{i}\left(a\right)$ in the MSA. Here, we show the histograms of Hamming distances from the consensus for natural sequences and artificial ones in the case of Pfam family PF00076; here the DCA method employed to generate artificial sequences is Adaptive Cluster Expansion (ACE) [37, 45], that accurately reproduces the sampled and correlation at the cost of a high computational demand. Hamming distances of natural and model-generated sequences from Pfam family PF00076. The two histograms show that, from the point of view of the Hamming distance, natural and artificial sequences are in fact indistinguishable.(TIF)

S1 File ID Pipeline. The archive contains the following files we used to run the ID analysis: (i) a readme file, (ii) a shell script file, (iii) a fasta file containing protein sequences, (iv) the main c++ file for the analysis.
(ZIP)
