# Supplementary figures and images for: The intrinsic dimension of protein sequence evolution
Source: PLoS Comput Biol. 2019 Apr 8;15(4):e1006767. doi: 10.1371/journal.pcbi.1006767 (PMC6472826; doi:10.1371/journal.pcbi.1006767)

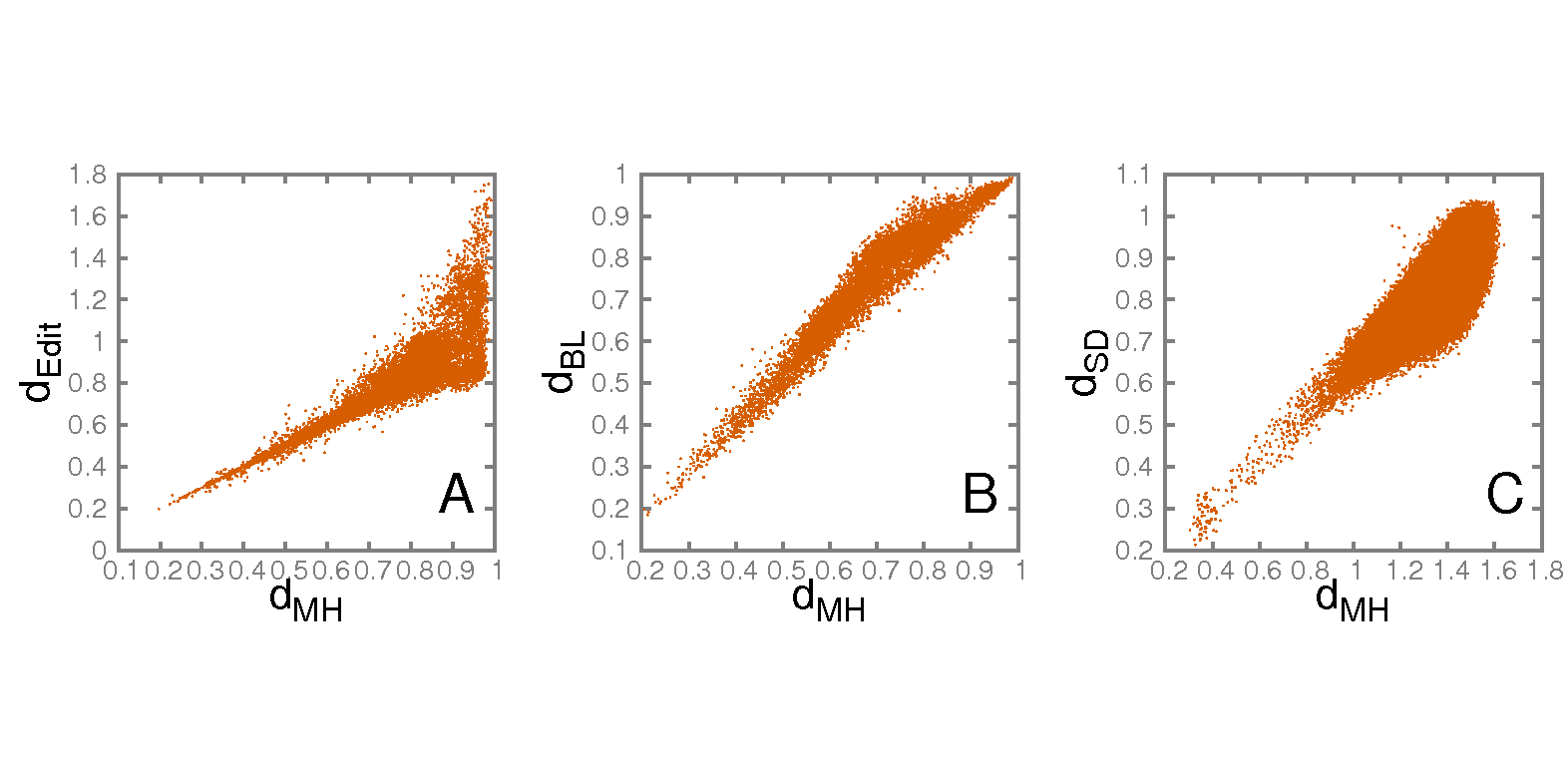

Supplement: S1 Fig — (A) Correlation plot between dBL and normalized Edit distances (B) Correlation plot between dBL and dMH distances. (C) Correlation plot between dSD and dMH distances. The four distances are correlated, especially at low values. (TIF) [file pcbi.1006767.s001.tif]

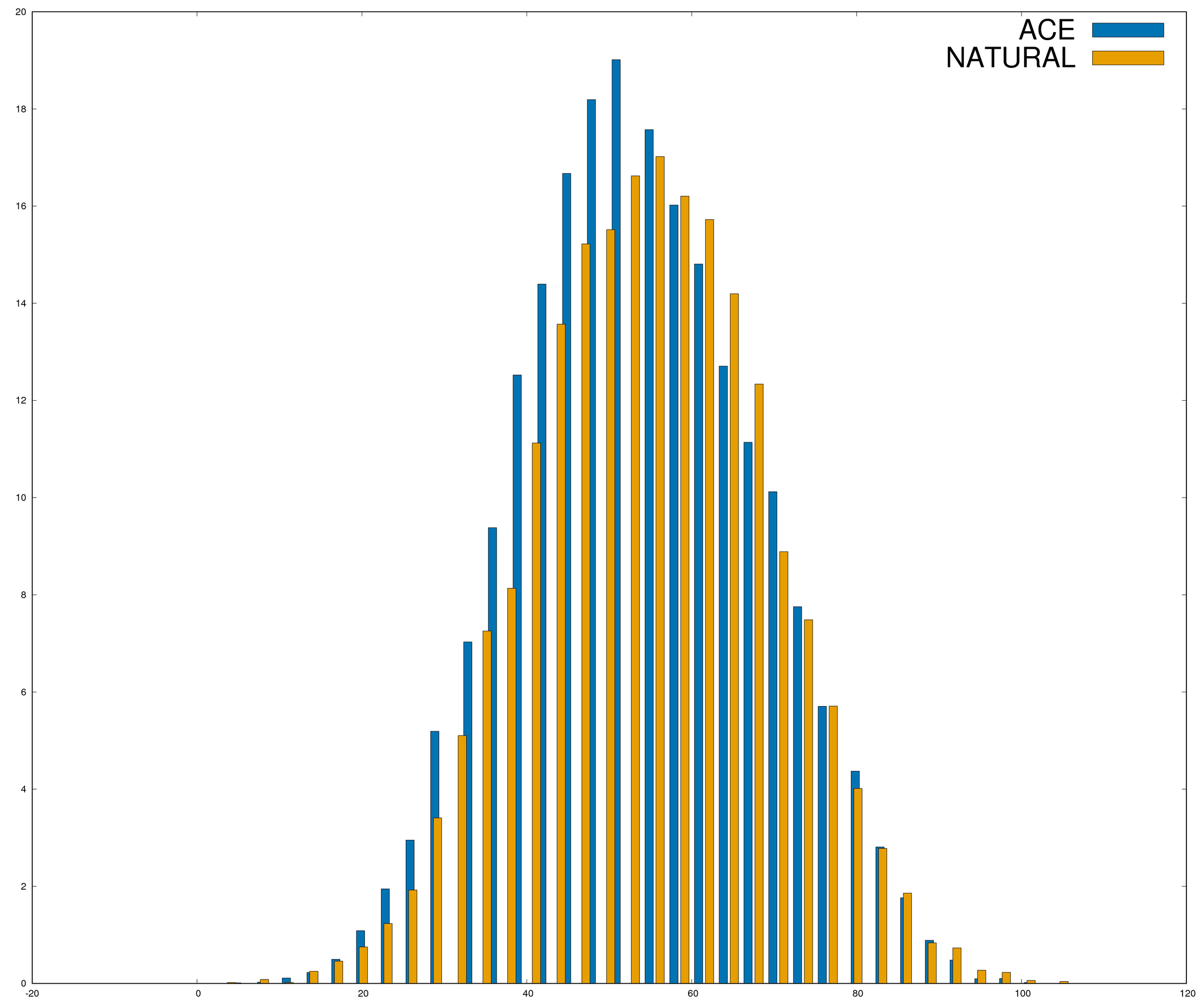

Supplement: S2 Fig — Artificial sequences should in principle be indistinguishable from natural ones. One of the characteristics they are supposed to share, tested in [14], is the set of Hamming distances of sequences to the consensus sequence (a1*,…,aL*), defined by the most frequent amino acids ai*=argmaxafi(a) in the MSA. Here, we show the histograms of Hamming distances from the consensus for natural sequences and artificial ones in the case of Pfam family PF00076; here the DCA method employed to generate artificial sequences is Adaptive Cluster Expansion (ACE) [37, 45], that accurately reproduces the sampled and correlation at the cost of a high computational demand. Hamming distances of natural and model-generated sequences from Pfam family PF00076. The two histograms show that, from the point of view of the Hamming distance, natural and artificial sequences are in fact indistinguishable. (TIF) [file pcbi.1006767.s002.tif]
